# Host-Parasite Relationship of Ticks (Acari: Ixodidae and Argasidae) and Feral Pigs (*Sus scrofa*) in the Nhecolândia Region of the Pantanal Wetlands in Mato Grosso do Sul

**DOI:** 10.5402/2013/610262

**Published:** 2013-05-19

**Authors:** P. H. D. Cançado, J. L. H. Faccini, H. M. Herrera, L. E. R. Tavares, G. M. Mourão, E. M. Piranda, R. C. S. Paes, C. C. D. U. Ribeiro, T. C. Borghesan, A. K. Piacenti, M. A. Kinas, C. C. Santos, T. M. Ono, F. Paiva

**Affiliations:** ^1^Embrapa Beef Cattle, Av. Rádio Maia 830, 79002-970 Campo Grande, MS, Brazil; ^2^Departamento de Parasitologia Animal, Universidade Federal Rural do Rio de Janeiro, 23890-000 Seropédica, RJ, Brazil; ^3^Universidade Católica Dom Bosco, 79117-010 Campo Grande, MS, Brazil; ^4^Centro de Ciências Biológicas e da Saúde, Universidade Federal de Mato Grosso do Sul, 79080-190 Campo Grande, MS, Brazil; ^5^Brazilian Agricultural Research Corporation-CPAP, Wild Life Laboratory, 79320-900 Corumbá, MS, Brazil; ^6^Agência Estadual de Defesa Sanitária Animal e Vegetal de Mato Grosso do Sul-IAGRO, 79074-902 Campo Grande, MS, Brazil; ^7^Programa de Pós-Graduação em Biologia da Relação Patógeno-Hospedeiro-ICB/USP, 05508-000 São Paulo, SP, Brazil; ^8^Programa de Pós-Graduação em Ciência Animal-UFMS, 79080-190 Campo Grande, MS, Brazil; ^9^Programa de Pós-Graduação em Ecologia e Conservação-UFMS, 79080-190 Campo Grande, MS, Brazil; ^10^Associação de Proprietários de RPPN do MS, 79002 004 Campo Grande, MS, Brazil

## Abstract

Feral pigs (*S. scrofa*) were introduced to the Pantanal region around 200 years ago and the population appears to be in expansion. Its eradication is considered to be impossible. The population of feral pigs in the Pantanal wetlands is currently estimated at one million. Two scientific excursions were organized. The first was conducted during the dry season, when 21 feral pigs were captured and the second was during the wet season, when 23 feral pigs were captured. Ticks were collected and the oviposition and hatching process were studied to confirm the biological success of each tick species. Three tick species were found to be feeding on feral pigs: *Amblyomma cajennense*, *A. parvum*, and *Ornithodoros rostratus*. During the dry season, 178 adult *A. cajennense* were collected, contrasting with 127 *A. cajennense* specimens in the wet season. This suggests that the seasonality of these ticks in the Brazilian Pantanal wetlands could be different from other regions. The results indicate that *A. parvum* and *A. cajennense* are biologically successful parasites in relation to feral pigs. *A. cajennense* appears to have adapted to this tick-host relationship, as well as the areas where feral pigs are abundant, and could play a role in the amplification of this tick population.

## 1. Introduction

Ticks have coevolved with various wild animal hosts which are reservoir hosts for pathogens such as fungi, bacteria, viruses, rickettsiae, and protozoan which can be transmitted to domestic mammals and humans [1–3].

Ticks that feed on feral or domestic pigs (*Sus scrofa*) and their tick-borne diseases have been previously studied worldwide [4–7]. The most common tick species reported in association with domestic pigs in Brazil is *Amblyomma cajennense* (Fabricius, 1787), an eclectic tick that has a broad range of hosts and widespread distribution [8–10]. In Brazil, this hard tick is an important vector of *Rickettsia rickettsii *to humans [11]. A second genus reported in association with Brazilian pigs is the genus *Ornithodoros*, which has two species: *O. rostratus* and *O. brasiliensis*. Both of these species have also been reported in association with wild native pigs (*Tayassu* sp.) [8, 9]. 

The Pantanal ecosystem is considered to be one of the most well-preserved biomes in Brazil and was added to the UNESCO World Heritage List in 2000. The Brazilian Pantanal has been described as a “biological hotspot” for conservation and one of the richest and the most diverse ecosystems in the world. In this biome, wild animals and their parasites engage in complex and dynamic interactions. However, this environment has been modified in the last two decades due to a large substitution of forested areas by exotic pasture to increase the livestock population [12]. 

Domestic pigs (*Sus scrofa*) were introduced to the Pantanal by European settlers around two hundred years ago. Some escaped and became wild, giving rise to the current feral pig population [13, 14]. This foreign species is considered one of the “world's worst invasive alien species” by the World Conservation Union [15]. The population of feral pigs is expanding and its complete elimination from the Pantanal is now considered impossible. The population is currently estimated at over one million animals, dispersed in 10,000 groups [16]. 

The ecoepidemiological impact caused by feral pigs and their associated parasites in the Pantanal wetlands is unknown. Feral pigs are the main hunting target and are effectively acting as a replacement species for the hunting of native wildlife in the Pantanal [17]. Furthermore, since the feral pig is the main species hunted by local people, close contact with pigs during this traditional hunting practice may increase disease transmission to humans [18]. This scenario exemplifies the classical “man-made” environment [19]. 

The Brazilian Pantanal is an important area for conservation purposes and the cattle ranching industry in Latin America. In spite of this importance to the Brazilian economy, the ecology of ticks and tick-borne pathogens that infect free-living mammals in this region is poorly known. The aim of the present study was to describe tick fauna from feral pigs in Nhecolândia, a subregion of the Brazilian Pantanal, and to seek information about, tick biology, sex ratio, and seasonality.

## 2. Materials and Methods

### 2.1. Study Area

The Nhecolândia sub-region has a tropical climate with weather conditions that are markedly seasonal. The wet season (November–April) is hot and humid while the dry season (May–October) is warm. During the wet season, many parts of open grassland change from terrestrial to aquatic habitats. In the dry season, the land dries out and only scarce pools, creeks, and some lakes remain. The physiognomy of the studied area is dominated by Cerrado “Sensu Lato” (savanna), patches of forest, grasslands, and shallow lake basins [12].

### 2.2. Captures

The feral pigs were captured alive with the help of local people in two different seasons. The first capture trip was in the dry season, between July and October of 2004, when 21 feral pigs were captured. The second trip was in the wet season, in January of 2005, when 23 feral pigs were captured. The pigs were captured by fence traps or by cowboys who used a lasso to catch them. All captured pigs were tranquilized (tiletamine and Zolazepam—Zoletil) prior to examination and tick collection. All trapping and handling procedures were conducted in accordance with the authorization of the Brazilian Environment Institute (IBAMA) (license no. 183/2005). 

### 2.3. Tick Collection

During the first trip, all of the ticks collected from each animal were placed into plastic flasks with 70% ethanol. During the second trip, engorged females were maintained in small plastic boxes with a humid hydrophilic cotton ball to allow oviposition and larval eclosion. Generally, these two life cycle parameters are good predictors of host adequacy in tick biology. The site of tick attachment in each pig was also recorded. 

### 2.4. Identification

Ticks were identified using published morphological keys for neotropical ticks [20–22] and by comparison with specimens housed in the tick collection of the *Instituto Butantan* (SP, Brazil), under the care of curator Dr. D.M. Barros-Battesti.

### 2.5. Statistical Analysis

Quantitative descriptors (prevalence and mean abundance) were calculated for each parasite species, with males and females recorded separately [23]. Possible differences between prevalence (number of parasitized feral pigs in a given group of captured feral pigs) and mean abundance (mean quantity of ticks collected from each feral pig) were tested for the two different seasons using Fisher's exact test (prevalence) and Student's *t*-test (mean abundance), with previously log(*X* + 1) transformed intensity of infestation data [24]. The ratio of the variance to the mean abundance (DI) and the index of discrepancy (*D*) were used to determine distribution patterns [25].

Discriminant analysis, based on the Mahalanobis distance, was used to find differences between the two seasons, to classify different groups of hosts and to identify which parasite species were responsible for these differences. Analyses were performed using square-root transformed intensity of infestation data [26].

## 3. Results

Three tick species were found to be feeding on feral pigs in the Nhecolândia region: *Amblyomma cajennense*, *A. parvum*, and *Ornithodoros rostratus*. In both seasons, at least one specimen of *Amblyomma* was collected from each feral pig captured. Only adult *Amblyomma* were found. During the dry season, a total of 178 specimens of *A. cajennese* (8.48 ± 4.36 ticks/host—59 female and 119 male specimens) and 12 specimens of *A. parvum* (0.57 ± 1.78 ticks/host—four female and eight male specimens) were collected. In the wet season, a total of 127 specimens of *A. cajennense* were collected (5.52 ± 3.2 ticks/host—49 female and 78 male specimens), as well as four females of *A. parvum* (0.17 ± 0.49 ticks/host) (Table 1). A sex ratio of 1 : 0.50 and 1 : 0.63 (males : females) was found for *A. cajennense* in the dry and wet seasons, respectively.

The values of prevalence, mean abundance, and mean intensity of infestations and their possible differences can be seen in Table 2. Both *Amblyomma* species exhibited the typical aggregated pattern of distribution (Table 3).

The ticks did not exhibit a preference for any region of the pig's body and were collected from the rostrum, head, ears, neck, thorax, back, and members. All *A. cajennense* (23) or *A. parvum* (5) engorged females laid eggs with good hatch (>90%).

**Figure 1:**
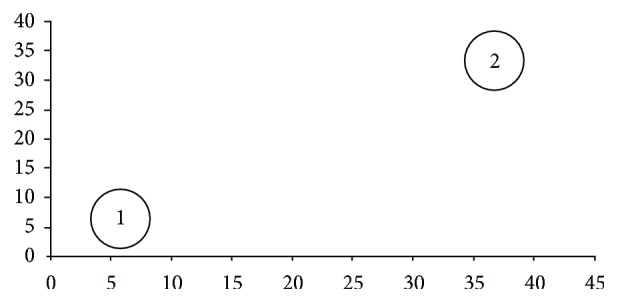
Sample scores of the first two discriminant axes for tick infracommunities of *Sus scrofa (feral pigs)* from the southern Pantanal wetlands in the State of Mato Grosso do Sul, Brazil. The numbers represent seasons: (1) wet season and (2) dry season, whereas the circles around the group represent the 95.7% tolerance region (e.g., 95.7% of the observations in a group are expected to lie inside the respective circle).

The first discriminant function explained 100% of the variance (eigenvalue = 0.33). Dimensionality tests for group separation revealed two distinct host groups (*χ* ^2^ = 11.31;  *P* = 0.02). A significant overall effect was observed (Wilk's lambda = 0.75; *F*_4,39_, *P* = 0.02). Each host specimen was 70.3% well classified in the two distinct groups (Table 4). The difference of prevalence and intensity for *A. cajennense *male infestation between the seasons was the most significant (95.7%) in determining the position of hosts among the groups (Figure 1).

**Figure 2:**
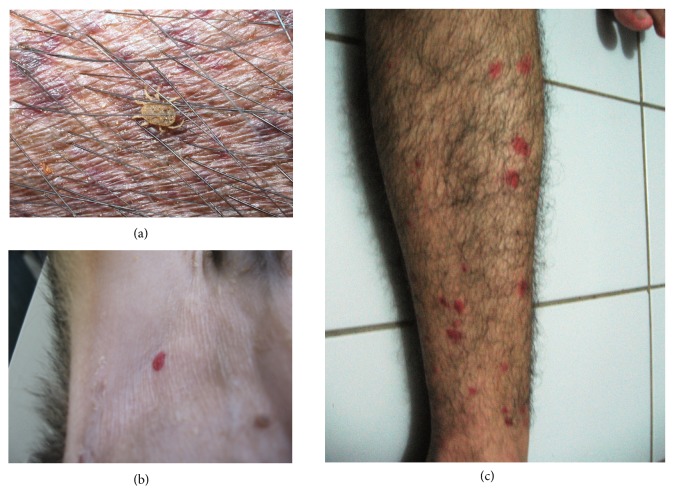
(a) Nymph of *O. rostratus* feeding on a feral pig (*Sus scrofa*) and ecchymosis-like lesions; (b) skin lesion (ecchymosis) caused by nymph of *Ornithodoros rostratus* feeding on a dog; (c) ecchymosis-like lesions caused by nymphs of *O. rostratus* feeding on a human leg.

*O*.* rostratus* was only collected on one occasion (4 nymphs) during the wet season, feeding on the belly region (Figure 2(c)). Three nymphs molted to adults (two females and one male) in the laboratory. These were experimentally fed to domestic pigs. The larvae obtained under laboratory conditions were identified as *O. rostratus* [27]. The ecchymosis-like lesions observed in experimentally infested domestic pigs were similar to those found in naturally infested feral pigs. Although *O. rostratus* was not found, similar lesions were observed on domestic dogs and humans in the studied area (Figures 2(a), 2(b) and 2(c)).

## 4. Discussion

The prevalence and intensity of *A. cajennense* infestation found to be feeding on feral pigs, based on the observation that all engorged females laid viable eggs with a good hatching percentage (>90%), strongly suggest that this introduced mammal species is a very adequate host for this hard tick in the southern Pantanal region. Considering that feral pigs represent one of the major biomass free-living mammals in the studied area, as well as the fact that *A. cajennense* is a multi-host parasite, its amplification through feral pigs may play an important role in the health conditions of local wildlife, domestic animals, and people. In fact, a number of mammal species commonly found in the southern Pantanal have previously been naturally infested by *A. cajennense*: the collared peccary (*Pecari tajacu*); the white-lipped peccary (*Tayassu pecari*); the giant anteater (*Myrmecophaga tridactyla*); the collared anteater (*Tamandua tetradactyla*); the coati (*Nasua nasua*); the capybara (*Hydrochaeris hydrochaeris*); the marsh deer (*Blastocerus dichotomus*); and the brown brocket deer (*Mazama gouazoubira*) [28–30]. The crab-eating fox (*Cerdocyon thous*), the ocelot (*Leopardus pardalis*), and certain small rodents are examples of other wild mammals that have been infested by *A. cajennense* in the same studied area (ongoing unpublished research). This situation is a very good example of man-made opportunities for neo-tropical tick development [19]. The same author describes the expansion of the cayenne tick's (*A. cajennense*) geographical range as a result of its association with feral pigs, which are a highly mobile host. 

The discriminant analysis confirmed that *A. cajennense* was more abundant in the Nhecolândia sub-region during the dry season. In total, 70% of the feral pigs examined were correctly classified according to the seasons, considering the distribution of male *A. cajennense* to be responsible for this difference. These results are not in accordance with previously published studies. According to a number of studies, adult *A. cajennense* are more prevalent during the wet season [29, 31–36] because the annual water cycle in the Pantanal region is unique and differs from other Brazilian regions. Since this research comprises only one dry or wet season, more research concerning *A. cajennense* seasonality should be conducted before any solid conclusion. 

The higher intensity of infestation of male *A. cajennense* in relation to females in naturally infested feral pigs was probably due to the longer parasitic period of males. This has previously been suggested by Pinter et al. [32].

The results of the present study also suggest that feral pigs seem to be a good host for *A. parvum* in the southern Pantanal because (a) the prevalence was 15% and (b) all engorged females that were collected from feral pigs laid viable eggs from which hatched larvae were used to start a laboratory colony. Considering the constant environmental modifications by human activities in the Pantanal region and the broad host range of *A. parvum*, which has been reported to be infesting cattle, wild carnivores, giant anteaters, and deer [29, 30, 37, 38], feral pigs might be also an important source of infestation in domestic animals and humans. The recent discovery of a novel spotted fever group, *Rickettsia *sp., in *A. parvum* ticks from Argentina [39] is a concern in the studied area. 

With regard to infestations by *O. rostratus*, adults were obtained from nymphs collected from naturally infested feral pigs. These partial results indicate that feral pigs can be a source of infestation for domestic animals in the region studied. 

This tick frequently bites humans. People who have been bitten reported fever, swollen lymph nodes, and an intensive itch. The role of *O. rostratus* as a vector of tick-borne pathogens is poorly understood, although this tick is considered to be a vector of spotted fever [40, 41]. 

The three tick species found in the present study, *A. cajennense*, *A. parvum*, and *O. rostratus*, are able to cross-infect wildlife, domestic animals, and humans. This hosts' multiplicity suggests the existence of a complex network in the eco-epidemiology of these ticks in the Pantanal region.

Finally, one of the main causes of disease emergence or reemergence is environmental alterations that increase the contact between wildlife and domestic animals and its consequent modification of epidemiological profiles [42]. These feral pigs are an alien species which became greatly abundant and share the same habitat as autochthone wildlife and livestock. The role of their associated tick species in disease epidemiology needs to be widely investigated as a major concern for cattle production and wildlife conservation. In conclusion, ticks related to feral pigs may become a significant problem for humans, wildlife, and the health of livestock in the Pantanal region.

## Figures and Tables

**Table 1:** Mean, standard deviation (SD), and number of adult *Amblyomma* ticks collected from feral pigs in the Nhecolândia sub-region of the Pantanal between July 2004 and January 2005.

Parasites	Wet season				Dry season			
	Males	Females	Total	Mean ± SD	Males	Females	Total	Mean ± SD
*A. cajennense *	78	49	127	5.52 ± 3.20	119	59	178	8.48 ± 4.36
*A. parvum *	0	4	4	0.17 ± 0.49	8	4	12	0.57 ± 1.78

**Table 2:** Prevalence (P), mean abundance (MA), and mean intensity of *Amblyomma cajennense* and *A. parvum* parasites feeding on *Sus scrofa* from *X* and *Y* in the state of Mato Grosso do Sul, Brazil. Possible differences are tested with Fisher's exact test (prevalence) and Student's *t-*test (mean intensity).

Parasites	Wet season			Dry season			Differences	
	P%	MA ± SD	MI ± SD	P%	MA ± SD	MI ± SD	*P *	*t *
*A. cajennense * ♂	65.2	2.13 ± 2.67	3.27 ± 2.69	95.2	5.67 ± 4.98	5.95 ± 4.94	0.02	2.89∗
*A. cajennense * ♀	95.7	3.39 ± 2.52	3.55 ± 2.46	85.7	2.81 ± 1.94	3.28 ± 1.67	0.33	0.86
*A. parvum* ♂	—	—	—	14.3	1.16 ± 2.67	2.67 ± 2.08	—	—
*A. parvum *♀	13	0.17 ± 0.49	1.33 ± 0.57	9.5	0.19 ± 0.68	2 ± 1.41	1	−0.09

∗Significance level of *P* < 0.05; ♀: female; ♂: male.

**Table 3:** Dispersion index (ID) and index of discrepancy (*D*) for *Amblyomma cajennense* and *A. parvum *feeding on *Sus scrofa* from *X* and *Y* in the state of Mato Grosso do Sul, Brazil.

Parasites	Wet season		Dry season	
	ID	*D *	ID	*D *
*Amblyomma cajennense* ♂	3.34	0.59	4.38	0.4
*A. cajennense* ♀	1.37	0.37	1.34	0.36
*Amblyomma parvum* ♂	—	—	3.54	0.86
*A. parvum *♀	1.39	0.85	2.42	0.89

♀: female; ♂: male.

**Table 4:** Discriminant analysis showing the number and percentage of well-classified feral pigs in the dry and wet seasons.

Season	Number of well-classified pigs		%
	Dry season	Wet season	
Dry season	**17** ∗	6	**74** ∗
Wet season	7	**14** ∗	**67** ∗

Total	24	20	**70** ∗

∗Correctly classified.

## References

[1] Parola P., Raoult D., Service M. W. (2001). Tick-borne typhuses. *Encyclopedia of Arthropod-Transmitted Infections of Man and Domesticated Animals*.

[2] Parola P., Raoult D. (2001). Ticks and tickborne bacterial diseases in humans: an emerging infectious threat. *Clinical Infectious Diseases*.

[3] Jongejam F., Uilemberg G. (2004). The global importance of ticks. *Parasitology*.

[4] Greiner E. C., Humphrey P. P., Belden R. C., Frankenberger W. B., Austin D. H., Gibbs E. P. (1984). Ixodid ticks on feral swine in Florida. *Journal of Wildlife Diseases*.

[5] Kleiboeker S. B., Scoles G. A., Burrage T. G., Sur J. H. (1999). African swine fever virus replication in the midgut epithelium is required for infection of Ornithodoros ticks. *Journal of Virology*.

[6] Labuda M., Nuttall P. A. (2004). Tick-borne viruses. *Parasitology*.

[7] De La Fuente J., Naranjo V., Ruiz-Fons F. (2004). Prevalence of tick-borne pathogens in ixodid ticks (Acari: Ixodidae) collected from European wild boar (*Sus scrofa*) and Iberian red deer (Cervus elaphus hispanicus) in central Spain. *European Journal of Wildlife Research*.

[8] Aragão H. B. (1936). Ixodidas brasileiros e de alguns países limitrophes. *Memórias do Instituto Oswaldo Cruz*.

[9] Evans D. E., Martins J. R., Guglielmone A. A. (2000). A review of the ticks (Acari, Ixodida) of Brazil, their hosts and geographic distribution-1. The state of Rio Grande do Sul, Southern Brazil. *Memorias do Instituto Oswaldo Cruz*.

[10] Labruna M. B., Camargo L. M. A., Schumaker T. T. S., Camargo E. P. (2002). Parasitism of domestic swine (*Sus scrofa*) by Amblyomma ticks (Acari: Ixodidae) on a farm at Monte Negro, Western Amazon, Brazil. *Journal of Medical Entomology*.

[11] Guedes E., Leite R. C., Prata M. C. A., Pacheco R. C., Walker D. H., Labruna M. B. (2005). Detection of Rickettsia rickettsii in the tick *Amblyomma cajennense* in a new Brazilian spotted fever-endemic area in the state of Minas Gerais. *Memorias do Instituto Oswaldo Cruz*.

[12] Harris M. B., Arcângelo C., Pinto E. C. T., Camargo G., Neto M. B. R., Silva S. M. (2006). Estimativa da perda de cobertura vegetal original na Bacia do Alto Paraguai e Pantanal brasileiro: ameaças e perspectivas. Natureza e Conservação. *Fundação O Boticário de Proteção à Natureza: Revista Brasileira de Conservação da Natureza*.

[13] Alho C. J. R., Lacher T. E., Mares M. A., Schmidly D. J. (1991). Mammalian conservation in the Pantanal of Brazil. *Latin American Mammalogy: Topics in History, Biodiversity, and Conservation*.

[14] Sicuro F. L., Oliveira L. F. B. (2002). Coexistence of peccaries and feral hogs in the brazilian pantanal wetland: an ecomorphological view. *Journal of Mammalogy*.

[15] (IUCN) World Conservation Union (2000). *100 of the World Worst Invasive Alien Species-A Selection from the Global Invasive Species Database. Invasive Species Specialist Group*.

[16] Mourão G. M., Coutinho M. E., Mauro R. A., Tomás W. M., Magnusson W. (2002). Levantamento aéreos de espécies introduzidas no Pantanal: porco ferais (porco monteiro), gado bovino e búfalos, 1rd. *Boletim de Pesquisa e Desenvolvimento*.

[17] Jean Desbiez A. L., Keuroghlian A., Piovezan U., Bodmer R. E. (2011). Invasive species and bushmeat hunting contributing to wildlife conservation: the case of feral pigs in a Neotropical wetland. *Fauna & Flora International Oryx*.

[18] Lourival R. F. F., Fonseca G. A. B., Valladares-padua C., Bodmer R. E. (1997). Análise da sustentabilidade do modelo de caça tradicional, no Pantanal da Nhecolândia, Corumbá, MS. *Manejo e Conservação de Vida Silvestre no Brasil*.

[19] Evans D. E. (2001). *Man-Made Opportunities for Neotropical Ectoparasites Ecology of Desert Environments*.

[20] Aragão H., Fonseca F. (1961). Notas de Ixodologia. VIII Lista e chave para os representantes da fauna ixodológica brasileira. *Memórias do Instituto Oswaldo Cruz*.

[21] Guimarães J. H., Tucci E. C., Barros-battesti D. M. (2001). *Ectoparasitos de Importância Veterinária*.

[22] Barros-Battesti D. M., Arzua M., Bechara G. H. (2006). *Carrapatos de Importância Medico-Veterinária da Região Neotropical: Um Guia Ilustrado para Identificação de Espécies*.

[23] Bush A. O., Lafferty K. D., Lotz J. M., Shostak A. W. (1997). Parasitology meets ecology on its own terms: margolis et al. revisited. *Journal of Parasitology*.

[24] Zar J. H. (1999). *Biostatistical Analysis*.

[25] Poulin R. (1998). *Evolutionary Ecology of Parasites*.

[26] Ludwig J. A., Reynolds J. F. (1998). *Statistical Ecology: A Primer on Methods and Computing*.

[27] Kohls G. M., Sonenshine D. E., Clifford C. M. (1965). The systematics of the subfamily Ornithodorinae (Acarina: Argasidae). II. Identification of the larvae of the Western Hemisphere and descriptions of three new species. *Annals of the Entomological Society of America*.

[28] Ito F. H., Vasconcellos S. A., Bernardi F., Nascimento A. A., Labruna M. B., Arantes I. G. (1984). Evidência sorológica de Brucelose e leptospirose e parasitismo por Ixodideos em animais silvestres do Pantanal Sul-Mato-Grossense. *Arquivos De Veterinária*.

[29] Pereira M. C., Szabó M. P. J., Bechara G. H. (2000). Ticks (Acari: Ixodidae) associated with wild animals in the Pantanal region of Brazil. *Journal of Medical Entomology*.

[30] Martins J. R., Medri I. M., Oliveira C. M., Guglielmone A. (2004). Ocorrência de carrapatos em Tamanduá-Bandeira (Myrmecophaga tridactyla) e Tamanduá-mirim (Tamanduá-Tetradactyla) na região do Pantanal Sul-Mato-Grossense, Brasil. *Ciência Rural*.

[31] Oliveira P. R., Borges L. M. F., Lopes C. M. L., Leite R. C. (2000). Population dynamics of the free-living stages of *Amblyomma cajennense* (Fabricius, 1787) (Acari: Ixodidae) on pastures of Pedro Leopoldo, Minas Gerais State, Brazil. *Veterinary Parasitology*.

[32] Pinter A., Labruna M. B., Faccini J. L. H. (2002). The sex ratio of *Amblyomma cajennense* (Acari: Ixodidae) with notes on the male feeding period in the laboratory. *Veterinary Parasitology*.

[33] Labruna M. B., Kasai N., Ferreira F., Faccini J. L. H., Gennari S. M. (2002). Seasonal dynamics of ticks (Acari: Ixodidae) on horses in the state of São Paulo, Brazil. *Veterinary Parasitology*.

[34] Labruna M. B., Amaku M., Metzner J. A., Pinter A., Ferreira F. (2003). Larval behavioral diapause regulates life cycle of *Amblyomma cajennense* (Acari: Ixodidae) in Southeast Brazil. *Journal of Medical Entomology*.

[35] Estrada-Peña A., Guglielmone A. A., Mangold A. J. (2004). The distribution and ecological “preferences” of the tick *Amblyomma cajennense* (Acari: Ixodidae), an ectoparasite of humans and other mammals in the Americas. *Annals of Tropical Medicine and Parasitology*.

[36] Szabó M. P. J., Castro M. B., Ramos H. G. C. (2007). Species diversity and seasonality of free-living ticks (Acari: Ixodidae) in the natural habitat of wild Marsh deer (Blastocerus dichotomus) in Southeastern Brazil. *Veterinary Parasitology*.

[37] Nava S., Mangold A. J., Guglielmone A. A. (2006). The natural hosts for larvae and nymphs of *Amblyomma neumanni* and *Amblyomma parvum* (Acari: Ixodidae). *Experimental and Applied Acarology*.

[38] Labruna M. B., Jorge R. S. P., Sana D. A. (2005). Ticks (Acari: Ixodida) on wild carnivores in Brazil. *Experimental and Applied Acarology*.

[39] Pacheco R. C., Moraes-Filho J., Nava S., Brandão P. E., Richtzenhain L. J., Labruna M. B. (2007). Detection of a novel spotted fever group rickettsia in *Amblyomma parvum* ticks (Acari: Ixodidae) from Argentina. *Experimental and Applied Acarology*.

[40] Monteiro J. L., Fonseca F., Prado A. (1932). Typho endêmico de São Paulo-VI. Pesquisas sobre a possibilidade da transmissão experimental do vírus por Ixodidae. *Brasil Médico*.

[41] Hoogstraal H. (1985). Argasid and nuttalliellid ticks as parasites and vectors. *Advances in Parasitology C*.

[42] Lafferty K. D. (1997). Environmental parasitology: what can parasites tell us about human impacts on the environment?. *Parasitology Today*.

